# Incidence of acute pancreatitis among hospitalized patients with dengue: A systematic review and meta-analysis

**DOI:** 10.1371/journal.pntd.0014304

**Published:** 2026-05-04

**Authors:** Yu Chen, Meng Shang, Shengping Dou, Xiaoxu Wang, Haoqiang Ji, Xiuping Song, Jun Wang, Qiyong Liu

**Affiliations:** 1 National Key Laboratory of Intelligent Tracking and Forecasting for Infectious Diseases, National Institute for Communicable Disease Control and Prevention, Chinese Center for Disease Control and Prevention, Beijing, China; 2 Department of Vector Control, School of Public Health, Cheeloo College of Medicine, Shandong University, Jinan, Shandong, China; WRAIR, UNITED STATES OF AMERICA

## Abstract

**Background:**

Acute pancreatitis (AP) is an under-recognized but clinically relevant complication of dengue fever (DF), associated with rapid clinical deterioration and increased risk of mortality. However, the true incidence of this condition remains uncertain.

**Methods:**

Following PRISMA 2020 guidelines, we systematically searched PubMed, Web of Science, Embase and China National Knowledge Infrastructure (CNKI) up to May 2025 for observational studies reporting AP among DF hospitalized patients. Eligible studies were identified according to predefined inclusion and exclusion criteria based on the Population, Intervention, Comparison, Outcome, and Study design (PICOS) framework. Two reviewers independently screened the literature and assessed study quality using the Newcastle–Ottawa Scale (NOS). Pooled incidence rates were estimated using random or common effect models depending on heterogeneity.

**Results:**

Eight observational studies from five countries, comprising 1,078 hospitalized patients with DF, were included. Among them, 135 were diagnosed with AP. The pooled incidence of AP complicating DF was 12.4% (95% *CI*: 10.5–14.4%). Subgroup analysis suggested a higher incidence in studies with ≥100 participants compared to smaller cohorts (13.5% vs. 8.3%, *P* = 0.03). No evidence of significant publication bias was detected.

**Conclusion:**

DF-associated AP represents a clinically important complication, given the global burden of dengue and the potential severity of AP. Our findings underscore the importance of considering pancreatic enzyme testing, supplemented by imaging when clinically warranted, in dengue patients with persistent or severe abdominal symptoms and/or features of severe dengue. Large-scale, multicenter prospective studies are warranted to establish the true incidence and case-fatality risk, thereby informing evidence-based prevention and management strategies.

## Author summary

Dengue fever (DF) is one of the fastest growing mosquito-borne diseases worldwide, affecting millions of people each year. Although most patients present with mild symptoms such as fever, rash, and body aches, dengue can also cause severe and sometimes unexpected complications. One such complication is acute pancreatitis (AP), a sudden inflammation of the pancreas that may lead to serious illness and even death. To better understand this link, we performed a systematic review and meta-analysis by combining data from eight studies across five countries. Among more than 1,000 hospitalized patients with laboratory-confirmed DF, approximately one in eight developed AP. This finding indicates that AP is a clinically important complication of DF that should not be overlooked.Our study highlights the need for heightened clinical vigilance for abdominal symptoms in dengue patients, with targeted pancreatic enzyme testing and imaging when clinically indicated in those with persistent or severe abdominal symptoms and/or features of severe DF patients. Early recognition and management of this complication may reduce the risk of poor outcomes. Larger, multicenter studies are still required to confirm the true burden and improve treatment strategies.

## 1. Introduction

Dengue fever (DF) is an acute mosquito-borne viral infection caused by the dengue virus (DENV) [1]. Over the past two decades, its global incidence has increased dramatically. Between 2000 and 2019, reported cases increased from over 500,000 to approximately 5.2 million annually [2]. Current estimates suggest that 3.9 billion people are at risk of infection, with transmission now established in more than 100 countries across Africa, the Americas, the Eastern Mediterranean, Southeast Asia, and the Western Pacific. The greatest burden occurs in Asia, accounting for nearly 70% of global cases [3,4]. The expansion of dengue transmission has been attributed to climate change, rapid urbanization, globalization, international travel, and insufficient vector control, which together have enabled the principal vectors, *Aedes aegypti* and *Aedes albopictus*, to extend their geographic range into previously non-endemic areas, including parts of Europe [5–8]. DF is now recognized as one of the most pressing global public health challenges. [9–12]. The clinical manifestations of dengue are diverse, ranging from asymptomatic or mild febrile illness to severe, life-threatening conditions such as dengue hemorrhagic fever (DHF) and dengue shock syndrome (DSS) [13,14]. Severe disease is frequently associated with plasma leakage, multiorgan dysfunction, and increased mortality, particularly in patients with underlying comorbidities [15–17]. Reported complications include shock, central nervous system involvement, myocarditis, acute hepatic failure, and acute kidney injury.

More recently, acute pancreatitis (AP) has been identified as a possible but often overlooked complication of DF [18,19]. Several mechanisms have been proposed to explain this association, including direct viral invasion of pancreatic tissue, immune-mediated inflammatory injury, and ischemia secondary to plasma leakage [20–22]. AP is a common gastrointestinal emergency characterized by a wide clinical spectrum, ranging from mild, self-limiting inflammation to severe disease with multiorgan failure and death [23,24]. When associated with DF, AP adds complexity to both diagnosis and management. Prompt recognition is critical, as dengue-associated AP has been linked to prolonged hospitalization and additional complications, including acute lung injury, massive pleural effusion, gastrointestinal bleeding, and acute kidney injury [21]**.** The development of AP further complicates the clinical scope, prognosis and management of DF. Timely diagnosis and management of DF complicated with AP can reduce mortality and morbidity [25,26]. Studies indicate that AP in DF patients is correlated with prolonged hospitalization and complications, such as acute lung injury, massive pleural effusion, gastrointestinal bleeding, and acute kidney injury. Since AP demands aggressive fluid resuscitation, DF patients may face a life-threatening risk of fluid overload [27]. Despite these concerns, systematic evidence regarding the incidence of AP in DF patients remains scarce. Published studies report highly variable estimates, with incidence rates ranging from 7.0% to 15.3% [28,29]. Such discrepancies likely reflect differences in study design, diagnostic criteria, sample size, and geographic context. The lack of reliable pooled evidence hinders both clinical risk stratification and public health planning.

To address this knowledge gap, we conducted a systematic review and meta-analysis of observational studies reporting AP in patients with laboratory-confirmed DF. Our objectives were to provide a more precise estimate of the pooled incidence of this complication, to identify potential sources of heterogeneity, and to clarify its implications for clinical management and disease burden. This work aims to inform clinicians, researchers, and policymakers by emphasizing the need for vigilance in detecting atypical complications of dengue and by highlighting areas where further research is urgently required.

## 2. Materials and methods

### 2.1. Search strategy

This systematic review and meta-analysis was conducted in accordance with the PRISMA 2020 guidelines. A comprehensive search of PubMed, Web of Science, Embase and China National Knowledge Infrastructure (CNKI) was performed up to May 2025 to identify all relevant studies reporting AP in patients with dengue fever (DF) (S1 Table).

### 2.2. Inclusion and exclusion criteria

Eligibility criteria were defined using the PICOS (Population, Intervention, Comparison, Outcomes, Study design) framework. Studies that met the following criteria were included in this meta-analysis: (i) Human observational studies, such as cross-sectional, case-control, or cohort studies, providing data on patients with DF and AP. (ii) Studies reporting the author’s name, publication date, country of origin, number of DF cases, and number or incidence of DF combined with AP. Exclusion criteria: (i) Conference abstracts, case reports, reviews, letters, and duplicate publications, case reports or case series, etc. (ii) Studies that did not provide information on the incidence rate of DF concurrent with AP or were unable to calculate this incidence rate. The search strategy incorporated both keywords and MeSH terms, with detailed specifications provided in **S1 Table**. The retrieval was conducted without restrictions on language, publication type, or year.

### 2.3. Selection process

Two reviewers independently screened records in a two-step process: initial screening of titles and abstracts followed by full-text review. The reference lists of eligible articles were also examined to identify additional studies. No restrictions were applied regarding language, publication year, or study setting. Any discrepancies between the two reviewers were resolved through discussion, and if consensus could not be reached, two additional reviewers were consulted for adjudication.

### 2.4. Data extraction and quality evaluation

Data extraction was performed by one reviewer not involved in the initial screening, and independently verified by a second reviewer. Any disagreements were resolved by consensus or consultation with a third reviewer. Extracted data included: first author, year of publication, country/region, study design, data source, sample size, patient demographics (age and gender), total number of DF patients, and number or proportion of DF patients with AP. In addition, we extracted study-level information on how AP was defined and ascertained, including whether the (revised) Atlanta criteria were explicitly stated, the reporting of typical abdominal pain, the pancreatic enzyme markers and any thresholds used (amylase and/or lipase), whether imaging was performed and the imaging modality (e.g., ultrasonography, CT), whether alternative causes were assessed/excluded (e.g., gallstones, alcohol use), and whether pancreatitis severity grading was reported. These diagnostic features are summarized across studies in S2 Table; items not explicitly reported were coded as “Not stated (NS)”. For studies with missing or unclear information, attempts were made to contact corresponding authors. The methodological quality of the included studies was assessed using the Newcastle–Ottawa Scale (NOS), which evaluates selection, comparability, and outcome assessment domains. Studies were categorized as high, moderate, or low quality based on their NOS scores. Two reviewers conducted the assessment independently, and discrepancies were resolved through discussion.

### 2.5. Statistical analysis

Meta-analysis was performed using R software (version 4.3.1). Pooled incidence proportion with corresponding 95% confidence intervals (*CIs*) were calculated. Between-study heterogeneity was assessed using the Cochran Q test (*P* < 0.05 considered significant) and the I² statistic. Random-effects models were used as the primary approach because clinical and methodological diversity across studies was anticipated. Subgroup analyses were conducted based on study region, sample size, study design, patient demographics, and study quality to explore sources of heterogeneity. Subgroup analyses were prespecified to explore potential sources of between-study variability and were considered exploratory; therefore, P values for tests of subgroup differences were adjusted for multiple comparisons using the Benjamini–Hochberg false discovery rate procedure, with Bonferroni adjustment reported as a conservative sensitivity analysis. Sensitivity analyses were then performed using a leave-one-out approach by sequentially excluding each study to assess the robustness of the pooled estimates. Publication bias was evaluated visually using funnel plots and statistically using Begg’s and Egger’s tests. All statistical tests were two-sided, and *P* < 0.05 was considered statistically significant unless otherwise stated.

## 3. Results

### 3.1. Literature search and study characteristics

**Fig 1:**
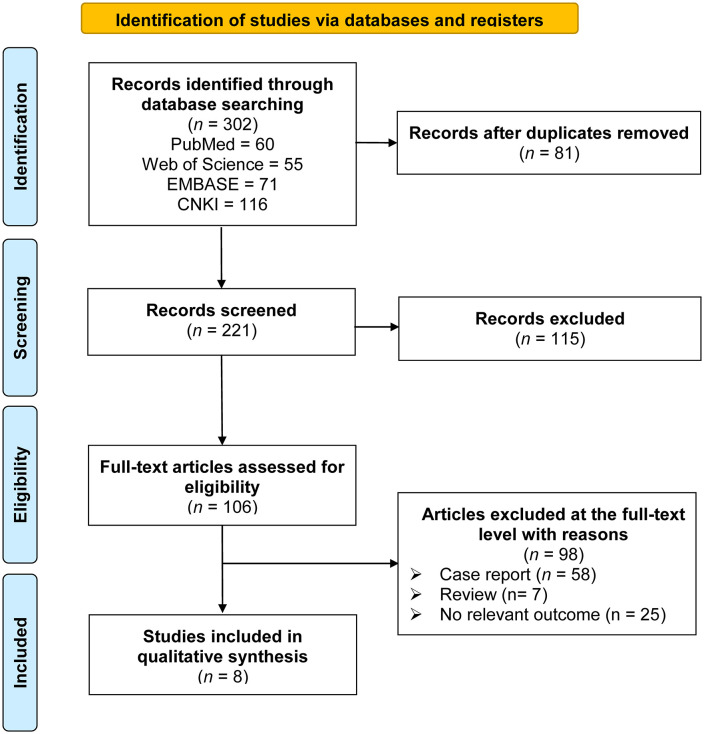
PRISMA flow diagram showing the process of study identification, screening, eligibility assessment, and inclusion.

The systematic search initially identified 302 potentially relevant records. After the removal of duplicates, 221 unique studies remained for screening. Title and abstract screening excluded 115 records that were clearly irrelevant, leaving 106 articles for full-text assessment. Of these, 98 studies were excluded for reasons such as insufficient data, inappropriate study design, or duplicate publication. Ultimately, 8 studies met the predefined eligibility criteria and were included in the quantitative synthesis. The study selection process is summarized in the PRISMA flowchart (Fig 1).

The characteristics of the included studies are shown in Table 1. Among the eight studies, four originated from India [28–31], while the others were conducted in Sri Lanka [32], China [33], Egypt [34], and Pakistan [26], respectively. In total, 1,078 hospitalized patients with laboratory-confirmed DF were included, of whom 135 were diagnosed with AP. All studies employed a cohort design, with four conducted prospectively and four retrospectively. The study populations demonstrated considerable demographic diversity, with participant ages ranging from 10 to 76 years and male representation varying from 35.0% to 90.0%. Methodological quality, as evaluated using the NOS, rated five studies as high quality and three as moderate quality. Notably, reporting of clinical outcome endpoints was limited: none of the included studies reported mortality, ICU admission, or length of hospital stay among DF patients with AP, and only one study provided pancreatitis severity grading.

**Table 1:** Characteristics of studies included in the meta-analysis (*n* = 8).

ID	First author (Year)	Country	Study design	Data source/ Study Setting	Age (years)	Sample size (DF)	Sample size (DF with AP)	Quality of literature
1	Khanna S. (2005) [30]	India	Prospective cohort study	Pushpawati Singhania Research Institute for Liver, Renal and Digestive Disease (Jul 2003–Nov 2004)	20–67 (mean 35.5)	55	8	High
2	Jayasundara B. (2017) [32]	Sri Lanka	Retrospective cohort study	National Hospital of Sri Lanka and Colombo South Teaching Hospital (Dec 2012–Dec 2013)	10 ~ 71	17	1	High
3	Ghweil AA. (2019) [34]	Egypt	Prospective cohort study	Qena University Hospital (Oct 2017–Apr 2018)	40.34 ± 15.74	100	13	Medium
4	Shamim M. (2010) [26]	Pakistan	Prospective cohort study	Three secondary care hospitals, Karachi (Jun 2005–Dec 2008)	13–72 (mean 29.9)	43	3	High
5	Mahajan V. (2024) [28]	India	Retrospective cohort study	Tertiary care hospital, North India (Jun 2021–Jun 2022)	3.7–13.5 (mean 9)	33	2	Medium
6	Mohanty B. (2019) [31]	India	Prospective cohort study	Department of Medicine, Tata Main Hospital, Jamshedpur, Jharkhand (Apr 2017–Mar 2018)	12–76 (mean 47.5)	520	62	High
7	Majumdar R. (2012) [29]	India	Retrospective cohort study	RG Kar Medical College and Hospital, Kolkata (2012 outbreak)	15–40 (mean 32.5)	300	45	High
8	Gan Liu (2020) [33]	China	Retrospective cohort study	Wannan region, imported DF cases (Jan 2018–Dec 2019)	28–45 (mean 47.5)	10	1	Medium

### 3.2. Overall incidence of DF complicated with AP

**Fig 2:**
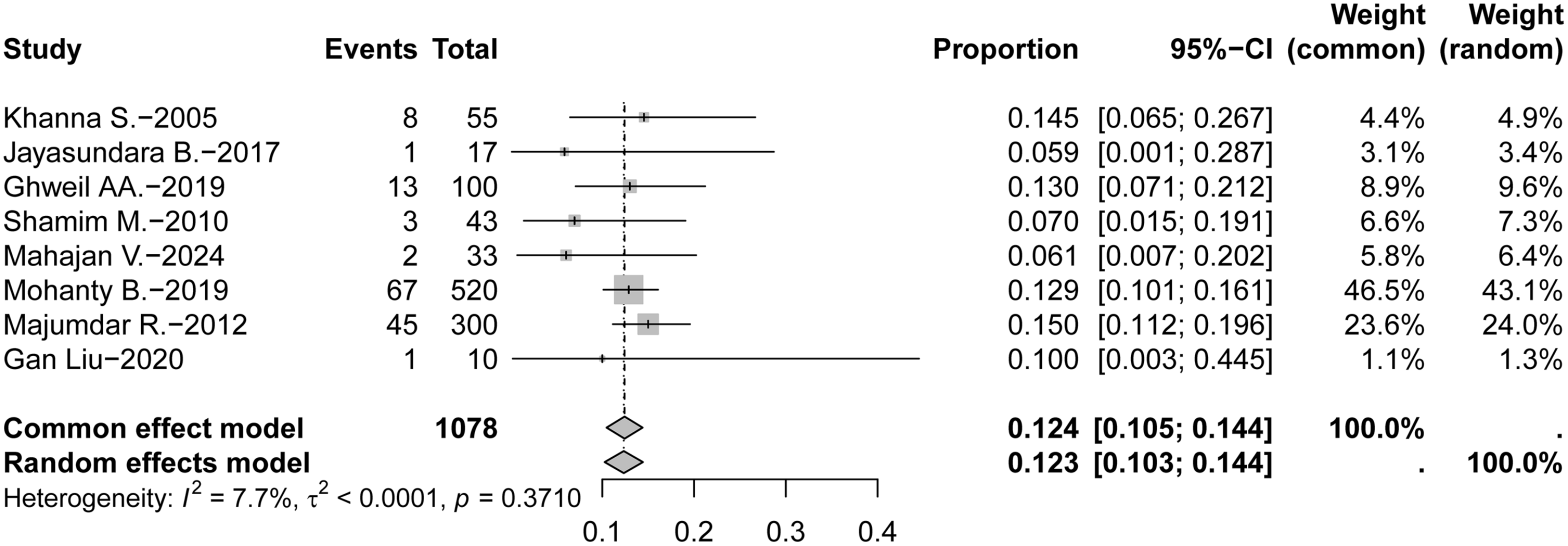
Forest plot showing the individual study estimates and pooled incidence of AP among hospitalized patients with DF.

Across the eight included studies, a total of 1,078 patients with laboratory-confirmed DF were reported, of whom 135 were diagnosed with AP. The reported incidence of AP among hospitalized DF patients ranged from 5.9% to 15.0%. Five studies documented an incidence exceeding 10%, while three reported rates below 10%. Assessment of heterogeneity revealed no significant variability among studies (Q = 7.58, *P* = 0.371; I² = 7.7%). A random-effects model was prioritized for the meta-analysis to account for the potential clinical and geographical diversity across the study populations (e.g., India, China, Egypt). Based on this model, the pooled incidence of DF complicated by AP was 12.4% (95% *CI:* 10.3 ~ 14.4%) (**Fig 2**).

### 3.3. Subgroup analyses

**Fig 3:**
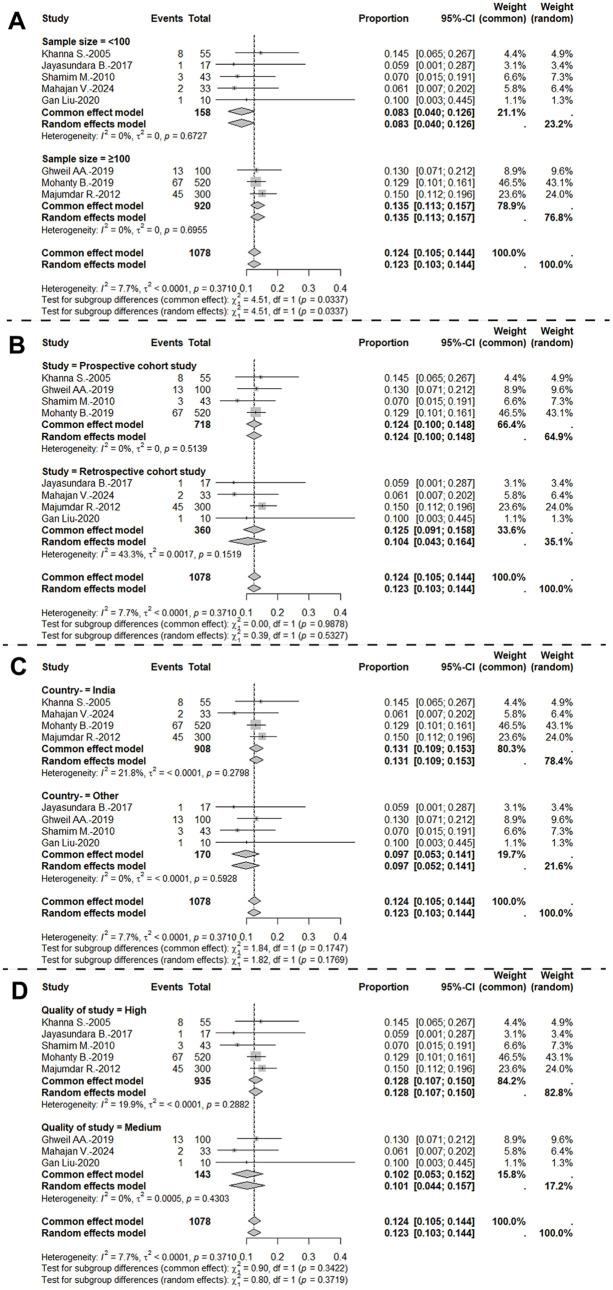
Subgroup analyses of the incidence of AP among hospitalized DF patients. Panels A ~ D present forest plots of subgroup analyses stratified by different study characteristics. **(A)** Sample size (<100 vs. ≥ 100 participants); **(B)** Study design (prospective vs. retrospective cohorts); **(C)** Study region (India vs. other countries); **(D)** Methodological quality (high vs. moderate Newcastle–Ottawa Scale score). Squares represent the effect size of each study, with the size proportional to study weight; horizontal lines denote 95% confidence intervals; and diamonds indicate pooled estimates within each subgroup.

To further investigate potential sources of heterogeneity, subgroup and meta-regression analyses were performed (**Fig 3**). Stratification by sample size revealed that studies enrolling fewer than 100 participants reported a pooled incidence of 8.3% (95% *CI*: 4.0 ~ 12.6%), whereas studies with ≥100 participants showed a higher pooled incidence of 13.5% (95% *CI*: 11.3 ~ 15.7%). Analysis by study region indicated that four studies conducted in India reported an incidence of 13.1% (95% *CI*: 10.9 ~ 15.3%), while studies from other countries collectively yielded an incidence of 9.7% (95% *CI*: 5.2 ~ 14.1%). When stratified by study design, prospective cohort studies reported an incidence of 12.4% (95% *CI*: 10.0 ~ 14.8%), compared with 10.4% (95% *CI*: 4.3 ~ 16.4%) in retrospective cohorts. Regarding study quality, high-quality studies documented an incidence of 12.8% (95% *CI*: 10.7 ~ 15.0%), while moderate-quality studies reported 10.1% (95% *CI*: 4.4 ~ 15.7%).

A nominal subgroup difference was observed for sample size (Q_between = 4.51, df = 1, *P* = 0.0337). However, because multiple subgroup comparisons were conducted, we applied a multiple-testing adjustment; after correction, the sample-size subgroup difference was no longer statistically significant (BH-adjusted *P* = 0.1348; Bonferroni-adjusted *P* = 0.1348). This finding should therefore be interpreted cautiously, as it may reflect detection/ascertainment differences, power imbalances, or correlation with study quality rather than true effect modification. No statistically significant subgroup differences were observed for location, study design, or study quality.

### 3.4. Publication bias

**Fig 4:**
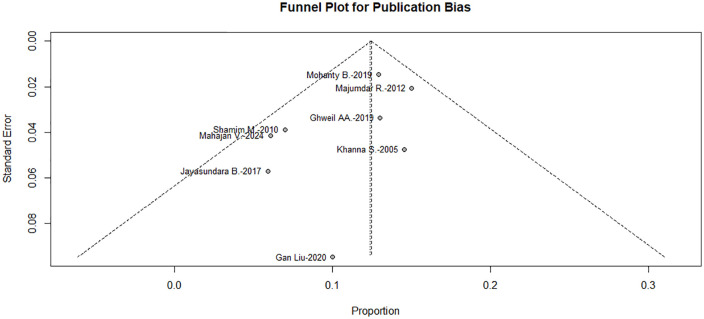
Funnel plot assessing potential publication bias among included studies.

Potential publication bias was evaluated using both visual and statistical methods. Inspection of the funnel plot suggested a relatively symmetrical distribution of studies (**Fig 4**). And formal tests did not detect statistically significant small-study effects: Begg’s test (Z = –1.10, *P* = 0.27), Egger’s test (t = –0.64, *P* = 0.54), and Harbord’s test (t = –0.85, *P* = 0.43). However, given the limited number of included studies (*n* = 8), these tests have low statistical power and should be interpreted with caution. Therefore, publication bias cannot be definitively excluded, although it is unlikely to have materially influenced the pooled results.

### 3.5. Sensitivity analysis

**Fig 5:**
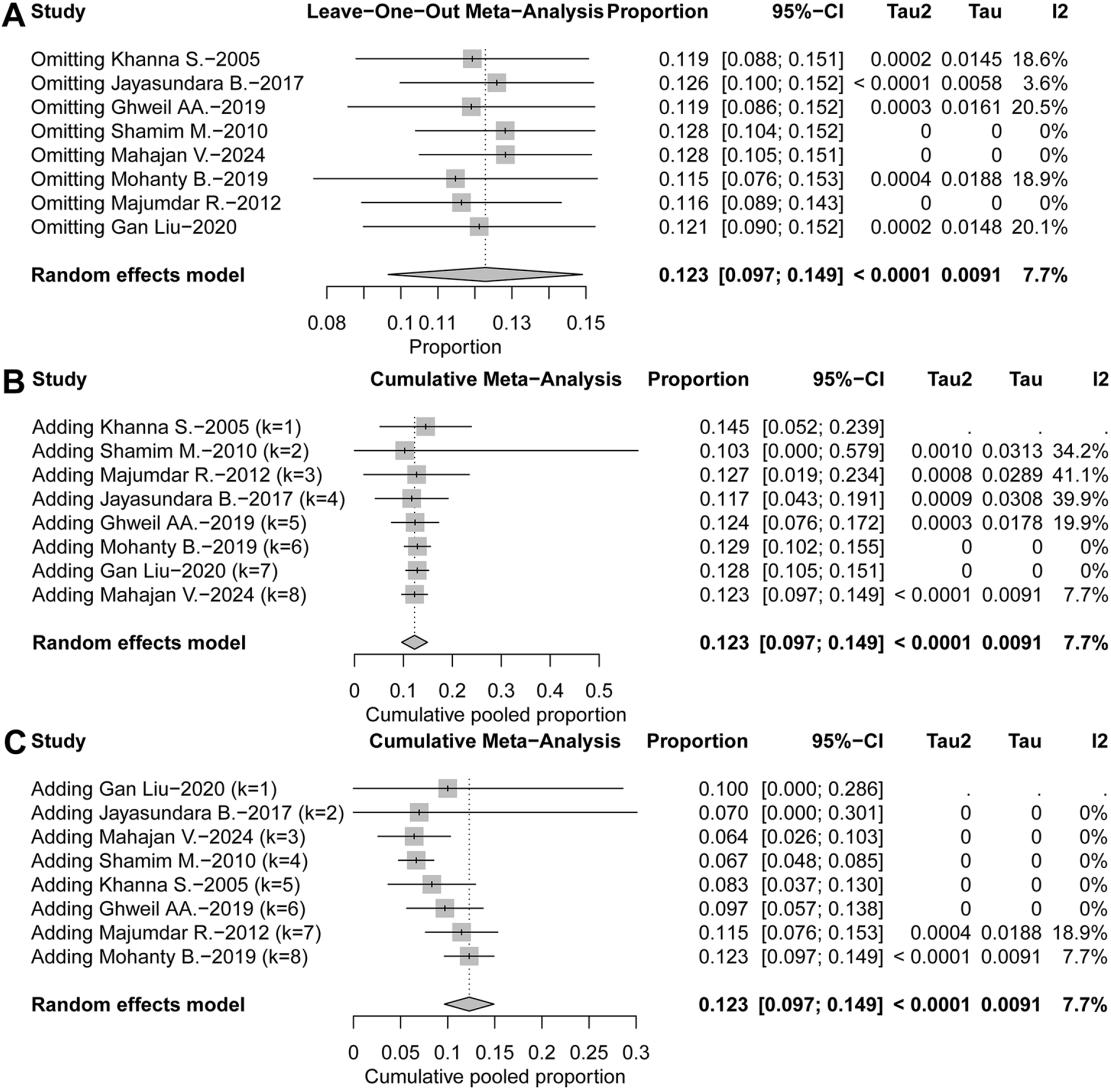
(A) Sensitivity analysis evaluating the stability of pooled incidence estimates through leave-one-out analyses.(B) Cumulative meta-analysis of the pooled proportion of AP among hospitalized DF patients, with studies sequentially added in order of publication year. (C) Cumulative meta-analysis with studies sequentially added in order of sample size.

Sensitivity analyses were conducted to assess the robustness of the pooled incidence estimate. Sequential exclusion of each study, one at a time, demonstrated that the overall pooled incidence of AP among DF patients remained stable and did not vary substantially with the omission of any single study (**Fig 5A**). To further evaluate the potential influence of larger cohorts, we performed cumulative meta-analyses. When studies were added sequentially by publication year, the pooled proportion evolved gradually and became more precise as evidence accumulated, without abrupt shifts in the point estimate (**Fig 5B**). A similar pattern was observed when studies were added in order of increasing sample size (**Fig 5C**). The final pooled estimate was 0.123 (95% *CI*: 0.097 ~ 0.149) with low heterogeneity (I² = 7.7%). These findings indicate that larger studies primarily improve precision, while the overall conclusion remains robust and is not driven by any single study.

## 4. Discussion

In recent years, scattered case reports and cohort studies from Brazil [20], India [35], Egypt [34] and China Taiwan [18] have documented dengue-associated AP, implying that the close link between the two conditions is not coincidental [19,24,35].These observations have raised concern that DF infection may trigger under-recognized pancreatic inflammation. This systematic review and meta-analysis provides the first pooled global estimate of AP incidence in hospitalized patients with DF. Based on eight observational studies including 1,078 laboratory-confirmed DF cases from five countries, the pooled incidence of AP was 12.4%.

Our findings highlight that AP is an under-recognized yet not rare complication among hospitalized DF patients, with meaningful clinical and public health implications in light of DF’ s substantial global burden and the potential severity of pancreatitis.. In endemic regions with millions of annual DF cases, even a modest incidence translates into a considerable number of AP cases globally [36,37]. Importantly, AP can complicate the clinical course of dengue, prolong hospitalization, and increase the risk of severe complications such as pleural effusion, gastrointestinal bleeding, and acute kidney injury [18,20,26,38]. Furthermore, management of AP often requires aggressive fluid resuscitation, which poses additional risks in DF patients prone to plasma leakage and fluid overload [25,39]. These challenges underscore the need for early recognition and timely management of this complication [27].

The subgroup analyses offer additional clues to the sources of between-study variation. Studies with larger sample sizes (≥100 patients) reported higher AP incidence compared to smaller studies, suggesting that small studies may underestimate the true burden due to limited statistical power or incomplete case ascertainment [40,41]. Regional analysis indicated that studies from India reported higher incidence rates compared to other countries, although this finding should be interpreted cautiously given the limited number of studies outside India. Similarly, prospective studies and those rated as high quality consistently reported higher incidence estimates than retrospective or moderate-quality studies. This pattern suggests that rigorous study design and diagnostic intensity may improve detection of atypical dengue manifestations such as AP. Importantly, even with low statistical heterogeneity, meaningful clinical and methodological differences likely remain across settings. Variation may arise from differences in dengue severity and hospitalization practices (which shape the inpatient case-mix), as well as inconsistent pancreatitis ascertainment (systematic vs symptom-triggered enzyme testing, imaging use, and application of standardized criteria such as the revised Atlanta classification). Differences in underlying host risk factors (e.g., gallstones, hypertriglyceridemia, alcohol use) and variable control of these confounders may further contribute. Given the small number of available studies, these explanations are hypothesis-generating, underscoring the need for multicenter studies using standardized diagnostic protocols to better define true cross-country variability.

The pathogenesis of dengue-associated AP is likely multifactorial. Proposed mechanisms include direct viral invasion of pancreatic acinar cells, immune-mediated injury, microvascular ischemia secondary to plasma leakage, and dysregulated host inflammatory responses [20,21]. Accumulating evidence suggests that AP represents an acute and atypical manifestation. AP typically arises during the viremic or early convalescent phase, implicating both direct cytopathic and delayed immunopathological mechanisms [19,36,38]. This temporal relationship implicates delayed immunopathological mechanisms in the development of late-onset AP. However, the role of age in the development and severity of dengue-associated AP remains unclear. Since DF presents a wide spectrum of clinical features with significant diagnostic implications, AP may emerge as a late complication, with potentially different manifestations in pediatric versus adult populations. Therefore, its early recognition through appropriate management strategies is essential to reduce morbidity and mortality [34,42]. Future research should include age-specific analyses to explore potential age-related differences in the incidence, clinical course, and outcomes of DF-associated AP.

From a public health perspective, our findings support the integration of AP monitoring into dengue management protocols, particularly in high-incidence regions. Routine testing of serum amylase and lipase, combined with appropriate imaging, may facilitate earlier diagnosis and intervention, thereby reducing preventable morbidity and mortality. Moreover, these results underscore the importance of developing standardized diagnostic criteria and follow-up protocols to ensure comparability across studies and improve clinical outcomes.

### 4.1. Limitations

This meta-analysis has several limitations. First, the majority of included studies were conducted in India, which may limit the generalizability of our findings to other regions. Second, the total number of eligible studies was relatively small, precluding comprehensive subgroup analyses by age, sex, comorbidities, or clinical severity. Third, data on mortality and long-term outcomes of dengue-associated AP were insufficient, highlighting the need for future research in this area. Fourth, the included cohorts were predominantly hospital-based, so the pooled estimate likely reflects the occurrence of AP among hospitalized managed DF patients rather than community-managed infections. This may introduce selection and referral bias, as admitted patients may be older, have more comorbidities, and/or present with more severe clinical manifestations than the overall DF population. Finally, diagnostic criteria for AP were heterogeneous and often incompletely reported (Supplementary S2 Table), including inconsistent use of the revised Atlanta framework and unclear requirements for enzyme thresholds and imaging confirmation. This raises the possibility of outcome misclassification (e.g., transient DF-associated hyperamylasemia or non-specific abdominal inflammation), and the pooled proportion should therefore be interpreted cautiously.

### 4.2. Implications and future directions

Future studies should use standardized and explicitly reported case definitions for AP (e.g., revised Atlanta criteria), including clear enzyme thresholds and the role of imaging, and systematically capture severity grading and hard clinical outcomes to improve comparability across cohorts and enable more reliable synthesis. Additionally, studies should differentiate between pediatric and adult populations to understand potential age-related differences in disease presentation and outcomes. Well-designed, multicenter prospective studies are needed to generate robust occurrence estimates, define prognostic factors and longer-term sequelae, and inform evidence-based management; incorporating pancreatitis surveillance into DF research frameworks could support refinement of clinical guidelines and public health policy.

## 5. Conclusions

In this systematic review and meta-analysis of eight observational studies encompassing 1,078 hospitalized patients, the pooled incidence of AP complicating DF was 12.4%. Although AP may be under-recognized in DF, it occurs with a non-negligible frequency among hospitalized patients and warrants heightened clinical attention in light of DF’ s substantial global burden and the potentially fatal course of severe pancreatitis. Our findings highlight the urgent need for: (i) targeted pancreatic enzyme testing in DF patients presenting with abdominal pain, (ii) harmonized diagnostic and reporting standards to improve comparability across regions, and (iii) large-scale, multicenter prospective studies to refine incidence estimates, identify prognostic factors, and guide targeted interventions. Such measures are critical for improving patient outcomes and reducing the global burden of this underrecognized but clinically significant complication.

## Supporting information

S1 Table Search history. This file provides the complete database search history used in this systematic review, including the databases searched, search terms/strings, date ranges, and any applied limits/filters.
(DOCX)

S2 Table Diagnostic criteria for AP across included studies. This table summarizes how AP was defined and ascertained in each included study, including whether the (revised) Atlanta criteria were explicitly stated, reporting of typical abdominal pain, pancreatic enzyme markers/thresholds (amylase and/or lipase), imaging use and modality (e.g., ultrasonography, CT), assessment/exclusion of alternative causes, and whether severity grading was reported.
(DOCX)

S1 Checklist PRISM 2020 Checklist. This file provides a completed PRISMA 2020 reporting checklist that maps each PRISMA item to the exact page/section (or supplement) where it is addressed in our manuscript, to document compliance with PRISMA 2020 and facilitate transparent reporting.
(DOCX)
