# Supplementary material for: Incidence of acute pancreatitis among hospitalized patients with dengue: A systematic review and meta-analysis
Source: PLoS Negl Trop Dis. 2026 May 4;20(5):e0014304. doi: 10.1371/journal.pntd.0014304 (PMC13155676; doi:10.1371/journal.pntd.0014304)
Supplement: S1 Table — This file provides the complete database search history used in this systematic review, including the databases searched, search terms/strings, date ranges, and any applied limits/filters. (DOCX) [file pntd.0014304.s001.docx]

Supplemental Table 1. Search history

| Search ID | Query | Results |
| --- | --- | --- |
| **PubMed (Date: 5/31/2025)** | | |
| #3 | #1 AND #2 | 60 |
| #2 | ((((((((((((((Dengue Fever) ) OR (Fever, Dengue)) OR (Classical Dengue)) OR (Classical Dengues)) OR (Dengue, Classical)) ) OR (Classical Dengue Fever)) OR (Classical Dengue Fevers)) OR (Dengue Fever, Classical)) OR (Break-Bone Fever)) OR (Break Bone Fever)) OR (Fever, Break-Bone)) OR (Breakbone Fever)) OR (Fever, Breakbone) | 32845 |
| #1 | ((((((((((((((((((Acute pancreatitis) OR (Pancreatitis, Acute)) OR (Acute Pancreatitides)) OR (Pancreatitides, Acute)) OR (Pancreatitis, Acute Edematous)) OR (Acute Edematous Pancreatitides)) OR (Edematous Pancreatitides, Acute)) OR (Edematous Pancreatitis, Acute)) OR (Pancreatitides, Acute Edematous)) OR (Acute Edematous Pancreatitis)) OR (Peripancreatic Fat Necrosis)) OR (Fat Necrosis, Peripancreatic)) OR (Necrosis, Peripancreatic Fat)) OR (Peripancreatic Fat Necroses)) OR (Pancreatic Parenchymal Edema)) OR (Edema, Pancreatic Parenchymal)) OR (Pancreatic Parenchymal Edemas)) OR (Parenchymal Edema, Pancreatic)) OR (Pancreatic Parenchyma with Edema) | 82594 |
| **Web of Science (Date: 5/31/2024)** | | |
| #3 | #1 AND #2 | 55 |
| #2 | (((((((((((((TS=(Dengue fever)) OR TS=(Dengue Fever)) OR TS=(Fever, Dengue)) OR TS=(Classical Dengue)) OR TS=(Classical Dengues)) OR TS=(Dengue, Classical)) OR TS=(Classical Dengue Fever)) OR TS=(Classical Dengue Fevers)) OR TS=(Dengue Fever, Classical)) OR TS=(Break-Bone Fever)) OR TS=(Break Bone Fever)) OR TS=(Fever, Break-Bone)) OR TS=(Breakbone Fever)) OR TS=(Fever, Breakbone) | 31149 |
| #1 | (((((((((((((((((((TS=(Acute pancreatitis)) OR TS=(Pancreatitis, Acute)) OR TS=(Acute Pancreatitides))OR TS=(Pancreatitides, Acute)) OR TS=(Pancreatitis, Acute Edematous)) OR TS=(Acute Edematous Pancreatitides)) OR TS=(Edematous Pancreatitides, Acute)) OR TS=(Edematous Pancreatitis, Acute)) OR TS=(Pancreatitides, Acute Edematous)) OR TS=(Acute Edematous Pancreatitis)) OR TS=(Peripancreatic Fat Necrosis)) OR TS=(Fat Necrosis, Peripancreatic)) OR TS=(Necrosis, Peripancreatic Fat)) OR TS=(Peripancreatic Fat Necroses)) OR TS=(Pancreatic Parenchymal Edema)) OR TS=(Edema, Pancreatic Parenchymal)) OR TS=(Pancreatic Parenchymal Edemas))OR TS=(Parenchymal Edema, Pancreatic)) OR TS=(Pancreatic Parenchyma with Edema)) OR TS=(Acute Pancreatitis) | 62524 |
| **Embase (Date: 5/31/2024)** | | |
| #3 | #1 AND #2 | 71 |
| #2 | 'dengue'/exp OR 'Dengue virus'/exp OR 'Dengue virus 1'/exp OR 'Dengue virus 2'/exp OR 'Dengue virus 3'/exp OR 'Dengue virus 4'/exp OR 'dengue rapid test'/exp OR 'dengue hemorrhagic fever'/exp OR 'severe dengue'/exp OR 'dengue shock syndrome'/exp OR 'dengue vaccine'/exp OR 'Dengue virus test kit'/exp | 7276 |
| #1 | 'acute pancreatitis'/exp OR 'acute hemorrhagic pancreatitis'/exp OR 'cerulein-induced acute pancreatitis'/exp OR 'experimental acute pancreatitis'/exp | 58781 |
| **China National Knowledge Infrastructure (Date: 5/31/2024)** | | |
| #3 | #1 AND #2 | 116 |
| #2 | “Dengue fever” OR “Dengue virus” OR “Dengue disease” OR “Dengue fever virus” OR “Dengue fever epidemic” OR “Dengue fever outbreak” OR “Dengue fever cases” OR “Dengue fever patients” OR “Dengue hemorrhagic fever” OR “Dengue epidemic” | 4776 |
| #1 | “Pancreatitis treatment” OR “Pancreatitis patients” OR “Pancreatitis model” OR “Pancreatitis surgery” OR “Severity of pancreatitis” OR “Pancreatitis nursing” OR “Pancreatitis inflammation” OR “Complications of pancreatitis” OR “History of pancreatitis” OR  “Pathogenesis of pancreatitis” OR “Acute pancreatitis” OR “Severe acute pancreatitis” OR “Patients with acute pancreatitis” OR “Patients with severe acute pancreatitis” OR “Acute severe pancreatitis” OR “Patients with severe pancreatitis” OR “Biliary pancreatitis” OR “Acute pancreatitis (AP)” | 49738 |
