# Supplementary material for: Incidence of acute pancreatitis among hospitalized patients with dengue: A systematic review and meta-analysis
Source: PLoS Negl Trop Dis. 2026 May 4;20(5):e0014304. doi: 10.1371/journal.pntd.0014304 (PMC13155676; doi:10.1371/journal.pntd.0014304)
Supplement: S2 Table — This table summarizes how AP was defined and ascertained in each included study, including whether the (revised) Atlanta criteria were explicitly stated, reporting of typical abdominal pain, pancreatic enzyme markers/thresholds (amylase and/or lipase), imaging use and modality (e.g., ultrasonography, CT), assessment/exclusion of alternative causes, and whether severity grading was reported. (DOCX) [file pntd.0014304.s002.docx]

| Supplementary Table 2. Diagnostic criteria for AP across included studies. | | | | | | | |
| --- | --- | --- | --- | --- | --- | --- | --- |
| Study (Author, Year) | Population/Setting | Atlanta criteria stated | Typical abdominal pain | Enzymes used | Imaging performed (type) | Alternative causes assessed/excluded | Severity grading. |
| Khanna S.  (2005) (1) | Hospitalized dengue. | NS. | NS. | NS. | X-ray, ultrasonography and CECT. | NS. | NS. |
| Jayasundara B.  (2017) (2) | Hospitalized dengue. | NS. | NS. | NS. | **Ultrasonography.** | **Had past episodes of alcohol related pancreatitis.** | NS. |
| Ghweil AA.  (2019) (3) | Hospitalized dengue. | Yes. | NS. | Raised serum amylase (>  600 U/L) and  raised serum lipase (>  550 U/L). | Abdominal CT. | NS. | **Mild focal acute pancreatitis (*n*=10) and severe acute pancreatitis (*n*=3).** |
| Shamim M.  (2010) (4) | Hospitalized dengue. | Yes. | Yes. | Raised **serum amylase.** | Abdominal ultrasonography, chest X-ray (erect posture)and CT scanning. | NS. | NS. |
| Mahajan V.  (2024) (5) | Hospitalized dengue. | NS. | Yes. | NS. | NS. | NS. | NS. |
| Mohanty B.  (2019) (6) | Hospitalized dengue. | Yes. | NS. | Raised serum amylase and lipase | **Ultrasonography.** | NS. | NS. |
| Majumdar R.  (2012) (7) | Hospitalized dengue | NS. | NS. | NS. | NS. | NS. | NS. |
| Gan Liu  (2020) (8) | Hospitalized dengue. | Yes. | Yes. | Raised **serum amylase (**  389 U/L**) and**  raised serum lipase (183 U/L). | Abdominal CT. | NS. | NS. |

**NS: Not stated.**

****Reference****

1. Khanna S, Vij J, Kumar A, Singal D, Tandon R. Etiology of Abdominal Pain in Dengue Fever. Dengue Bulletin. 2005;29.

2. Jayasundara B, Perera L, de Silva A. Dengue fever may mislead the surgeons when it presents as an acute abdomen. Asian Pac J Trop Med. 2017;10(1):15-9.

3. Ghweil AA, Osman HA, Khodeary A, Okasha A, Hassan MH. Relative frequency of acute pancreatitis from dengue outbreaks as a late complication, in Egypt. Virusdisease. 2019;30(4):498-503.

4. Shamim M. Frequency, pattern and management of acute abdomen in dengue fever in Karachi, Pakistan. Asian J Surg. 2010;33(3):107-13.

5. Mahajan V, Singh J, Guglani V. CLINICAL PROFILE OF EXPANDED DENGUE SYNDROME IN CHILDREN. Pediatr Infect Dis J. 2024.

6. Mohanty B, Sunder A, Pathak S. Clinicolaboratory profile of expanded dengue syndrome - Our experience in a teaching hospital. J Family Med Prim Care. 2019;8(3):1022-7.

7. Majumdar R, Jana CK, Ghosh S, Biswas U. Clinical spectrum of dengue fever in a tertiary care centre with particular reference to atypical presentation in the 2012 outbreak in Kolkata. J Indian Med Assoc. 2012;110(12):904-6.

8. Liu G, Hu T, Li l, Wang W, Yang J, Sun E. Analysis of Epidemiological and Clinical Characteristics of 10 Cases of Imported Dengue Fever in Anhui Nan Region. Journal of Shenyang Medical College. 2020;22(05):408-11.
